# Lead aVR predicts early revascularization but not long-term events in patients referred for stress electrocardiography

**DOI:** 10.1371/journal.pone.0249779

**Published:** 2021-04-08

**Authors:** Aparna Baheti, Christopher A. Hanson, Michael McArdle, Sumeet K. Lall, George A. Beller, Jamieson M. Bourque

**Affiliations:** 1 University of Virginia School of Medicine, Charlottesville, VA, United States of America; 2 Division of Cardiovascular Medicine, Department of Medicine, The Cardiovascular Imaging Center, University of Virginia Health System, Charlottesville, VA, United States of America; Policlinico Casilino, ITALY

## Abstract

**Background:**

Exercise stress electrocardiography (ExECG) is recommended as a first-line tool to assess ischemia, but standard ST-analysis has limited diagnostic accuracy. ST elevation in lead aVR has been associated with left main and LAD disease in the population undergoing coronary angiography but has not been studied in the general population undergoing stress testing for the initial evaluation of CAD without coronary angiography. We sought to determine the predictive value of lead aVR elevation for ischemia, early revascularization, and subsequent cardiac events in consecutive patients undergoing ExECG.

**Methods and results:**

The study cohort included 641 subjects referred for ExECG who were dichotomized by presence or absence of aVR elevation ≥1mm and compared for prevalence and predictors of ischemia and a composite of cardiac death, nonfatal myocardial infarction, and late revascularization. The cohort had a median age of 57 and 57% were male. The prevalence of aVR elevation was 11.5%. The prevalence of significant ischemia on patients who received imaging was significantly higher with aVR elevation (14.3% vs 2.3%, p<0.001). Early revascularization occurred in 10.9% with vs 0.2% without aVR elevation, p<0.001. No subjects without aVR elevation or ST-depression underwent early revascularization. However, cardiac event rates were similar over a median 4.0 years of follow-up with and without aVR elevation (2.8% vs. 2.6%, p = 0.80). aVR elevation did not predict long-term cardiac events by Kaplan-Meier survival analysis (p = 0.94) or Cox proportional hazards modeling (p = 0.35).

**Conclusions:**

aVR elevation during ExECG predicts ischemia on imaging and early revascularization but not long-term outcomes and could serve as a useful adjunct to standard ST-analysis and potentially reduce the need for concurrent imaging.

## Background

Coronary artery disease (CAD) is a significant source of morbidity and mortality [1]. Exercise stress electrocardiography (ExECG) is an inexpensive, non-invasive diagnostic tool that is useful in the assessment of cardiovascular risk [2]. It is the first line test to risk-stratify patients with an intermediate risk of ischemic heart disease who have a normal resting ECG and are able to exercise [3]. Unfortunately, the low sensitivity of this test is a limitation. In a meta-analysis of patients with an intermediate risk of CAD, the sensitivity of ExECG was 68 percent in 132 studies of over 24,000 patients [4]. Accordingly, many patients are referred directly for noninvasive stress imaging to decrease the risk of a false negative study. In the recent PROMISE trial performed in major academic centers, ExECG was used alone in only 10% of patients evaluated for ischemia [5]. However, routine addition of imaging is suboptimal due to the additional study time, patient effort, economic impact, and radiation risk to the patient [6]. Additional strategies that improve the diagnostic accuracy of ExECG would encourage more appropriate use of this modality.

Lead aVR has shown promise in the diagnosis of CAD and as a prognostic marker for abnormal perfusion and cardiac events. It is uniquely positioned as a “pseudo-intracavitary” lead, allowing it to signal changes that may not be visible in other leads [7]. Prior studies have shown aVR elevation to predict high grade left main or ostial left anterior descending artery stenosis and an increased risk of cardiac events in the setting of an acute coronary syndrome [8, 9] and during stress testing [7, 10]. However, these studies examining diagnostic accuracy and prognosis have been primarily limited to patients undergoing invasive coronary angiography and were analyzed retrospectively. There have been multiple calls for evaluation of the diagnostic and prognostic significance of lead aVR in the general population undergoing an initial evaluation for CAD–as the initial evaluation for CAD does not normally include coronary angiography [11, 12].

Accordingly, we examined the prevalence of ≥1mm elevation of lead aVR during ExECG and assessed its diagnostic and prognostic impact and incremental value compared with clinical variables and stress myocardial perfusion imaging (MPI) or stress echocardiography in an unselected and consecutive cohort of outpatients referred for stress testing for their initial ischemia evaluation.

## Methods

We performed an analysis of a study cohort using previously-collected prospective data from the University of Virginia Stress Laboratory (Charlottesville, Virginia) to which outcomes data were subsequently collected and added.

### Study cohort derivation

**Fig 1:**
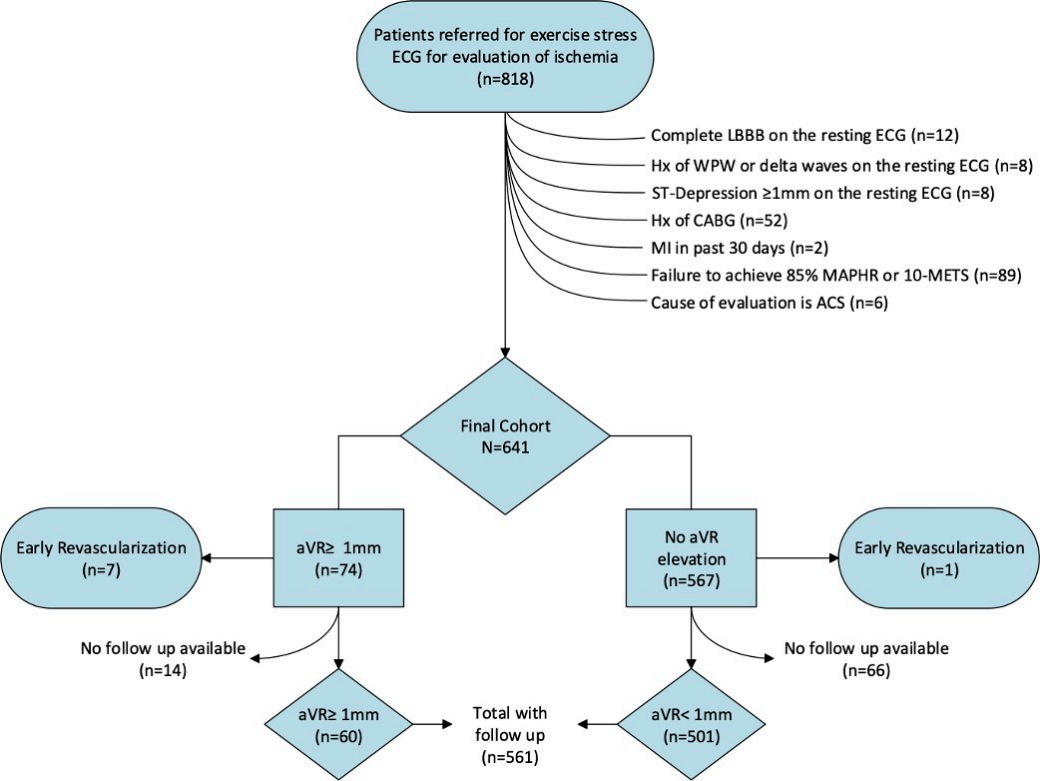
Patient flow diagram depicting the derivation of the study cohort.

The derivation of the study cohort is illustrated in Fig 1. We evaluated 818 consecutive outpatients referred for ExECG with or without imaging at the University of Virginia between July, 2007 and January, 2008 for the detection of myocardial ischemia. Patients were excluded if they had a complete left bundle branch block, ST-depression or elevation ≥1mm in any lead, or delta waves on the resting ECG, a history of WPW syndrome, a myocardial infarction (MI) in the preceding 30 days, prior coronary artery bypass surgery (CABG), or were under current evaluation for an acute coronary syndrome or had an elevated troponin-I >0.1 ng/dL. Subjects failing to achieve adequate stress, defined as ≥85% of their maximum age-predicted heart rate (MAPHR) or ≥10 metabolic equivalents (METS) of cardiac workload, were also excluded. Pharmacologic stress test subjects were excluded. The final study cohort included 641 subjects who were divided into 2 groups based on the presence or absence of ≥1mm elevation in lead aVR during ExECG.

### Clinical information collection and management

Clinical information collected at the time of ExECG included patient demographics, comorbidities, and physical examination and baseline ECG findings. The parameters of the exercise test as well as the stress imaging results (when applicable) were also collected [13]. Information on the presence of obstructive CAD and whether revascularization was performed was gathered on those patients who underwent invasive coronary angiography.

Follow-up data on all-cause and cardiac mortality and nonfatal cardiac events were obtained through chart review by a clinician blinded to the stress results, with questionable events verified by a second blinded clinician. For those patients in whom appropriate follow-up data could not be ascertained, a phone call was made to the patient to verify their health status. Protocol and follow-up questionnaire approval and waiver of informed consent were obtained from the University of Virginia Institutional Review Board.

### Exercise stress electrocardiography

**Fig 2:**
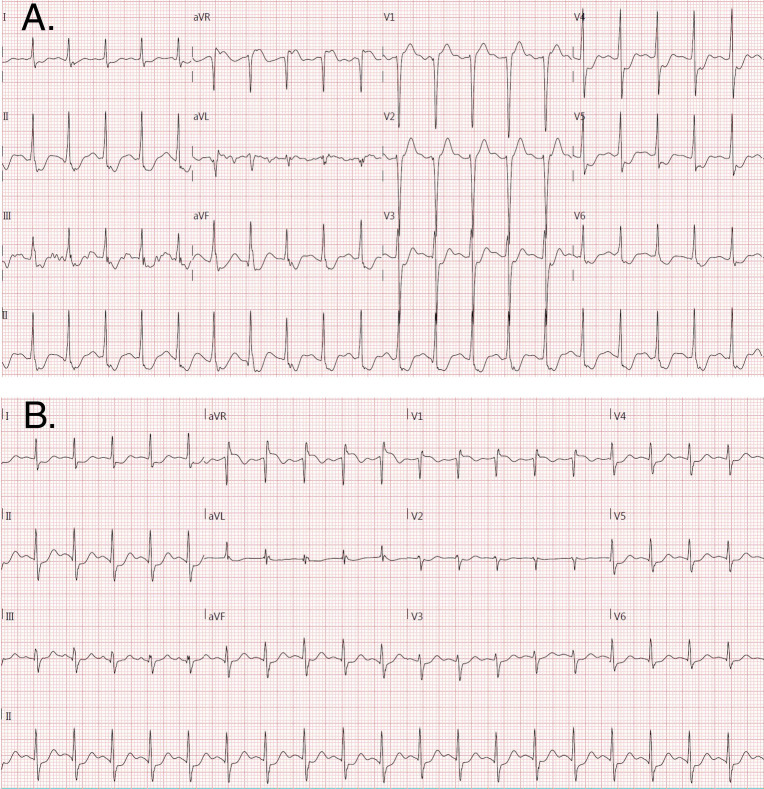
Examples of aVR elevation during stress electrocardiography with (A) and without (B) ST depression in lead V5. Note the subtle ST depression in tracing B does not meet criteria for ST depression on stress electrocardiography (defined as ≥1mm horizontal or down-sloping depression of the ST segment ≥80ms after the J-point for 3 consecutive beats).

ExECG was performed in accordance with the most recent ACC/AHA guidelines on exercise testing [3]. A standard or modified Bruce protocol was used in 99% of cases. Anti-ischemic medications were held at the discretion of the referring physician. The stress test was symptom-limited in the absence of ACC/AHA guideline-defined high-risk markers mandating early cessation [3]. All studies were interpreted by experienced electrocardiographers. Significant ST segment depression during ExECG was defined as ≥1mm horizontal or down-sloping depression of the ST segment ≥80ms after the J-point for 3 consecutive beats. Significant lead aVR elevation was defined as ≥1mm elevation from baseline ≥80ms after the J-point for 3 consecutive beats. Examples of lead aVR elevation with and without ST depression are included in Fig 2. Exercise workload was defined as the total METs achieved. The Duke Treadmill Score (DTS) was calculated using exercise time, maximum ST segment deviation, and exercise angina category as previously described [14]. The DTS was divided into low (DTS ≥5), moderate (DTS -10 to 4), and high (DTS <-10) risk tertiles as defined previously [15].

### Ischemia imaging assessment

A subset of the patients underwent subsequent imaging to assess for ischemia either with SPECT myocardial perfusion imaging (MPI) or stress echocardiography. Tc-99m Sestamibi rest-stress gated SPECT MPI was performed in accordance with the latest guidelines with standard dosages under a one-day protocol or over two days for those with a body mass index ≥36 [16]. The isotope was injected 1 minute prior to exercise cessation. Images were acquired with a dual-head GE Infinia camera with low-energy, high-resolution collimators using a standard Tc-99m energy window as per ASNC guidelines [16].

Nuclear cardiology specialists performed visual and quantitative image analysis [13]. Borderline and abnormal studies were verified by consensus opinion of two experienced readers blinded to the results of the stress ECG. A representation of the extent and severity of left ventricular (LV) inducible ischemia was derived using a standard 17-segment model [17]. Each segment was given a score from 0–4, with 0 representing normal perfusion and 1–3 representing mild, moderate, and severe defects respectively. A score of 4 signified absent tracer uptake. The semi-quantitative summed rest, stress, and difference scores were derived from these measurements. The percent myocardial ischemia, an indicator of both extent and severity of LV inducible ischemia, was calculated by dividing the difference between the summed stress and summed rest scores by the greatest possible difference, 68.

Stress echocardiography was performed in accordance with guidelines from the American Society of Echocardiography [18]. Left ventricular endocardial excursion and wall thickening were qualitatively assessed at rest and at peak stress by experienced echocardiographers using a standard 16-segment model. A new or worsening wall-motion abnormality in any of the 16 myocardial segments signified myocardial ischemia.

### Invasive coronary angiography

In patients referred, invasive coronary angiography was performed and interpreted by experienced operators with the assistance of computer-assisted quantitation (AGFA Heartlabs; Greenville, SC). Stenoses of ≥70% or equivocal stenoses of 50–69% with a fractional flow reserve of <0.8 were considered significant. Operators had access to all clinical data during study interpretation and for revascularization consideration.

### Outcomes

The primary outcome for this study was a composite of cardiac death, nonfatal MI, and late revascularization. Each of these was separately assessed as a secondary outcome. Cardiac death was defined as death with any demonstrable cardiac cause or without a clear non-cardiac cause. Non-fatal MI was defined as presentation of the patient consistent with acute coronary syndrome and a troponin ≥ 2 times the upper limit of normal, with or without typical ischemic changes on an ECG, which we defined as ≥1mm horizontal or down-sloping depression of the ST segment. Invasive coronary angiography and revascularization were considered late events when performed >4 weeks after ExECG.

### Statistical analysis

Continuous variables were described as medians with 25^th^ and 75^th^ percentiles and were compared with t-tests. Categorical variables were given as percentages and were compared with chi-square or Fisher’s exact testing where appropriate. Univariable logistic regression was performed to assess predictors of early revascularization. Multivariable logistic regression was performed on the two variables with the highest univariable Chi-square values due to constraints in the number of events. Univariable Cox proportional hazards analysis was performed to assess predictors of cardiac events. These predictors were then entered into a stepwise multivariable Cox model. A p<0.10 was required to enter the model, <0.05 to remain in the model. aVR elevation was forced into this model. Incremental chi-square analysis was performed predicting events using Cox proportional hazards analysis. Survival curves were generated using the Kaplan-Meier method [19]. The alpha level of significance for all analyses was 0.05. Statistical analysis was performed using SAS version 9.3 (SAS Institute, Cary, NC) and MedCalc version 12.5 (MedCalc Software, Ostend, Belgium).

## Results

### Study cohort characteristics

The clinical characteristics of the study cohort are provided in Table 1 in total and subdivided by presence or absence of aVR elevation ≥1mm. aVR elevation ≥1mm was present in 74 of 641 patients (11.5%). The group with aVR elevation was older and had a higher prevalence of hypertension, hyperlipidemia, and known CAD. Those without ≥1mm aVR elevation were more likely to have an elevated body mass index ≥30 kg/m^2^. There were no differences in gender, diabetes, tobacco use, history of MI, or prior revascularization between the groups.

**Table 1:** Clinical characteristics of the study cohort stratified by presence or absence of ≥1mm aVR elevation.

Characteristic	Entire Cohort	aVR Elevation ≥1mm	No aVR Elevation	P-value
	(n (%))	(n (%))	(n (%))	
**Patient Number**	641	74	567	-
Age*	57 (49, 67)	65 (57, 72)	56 (49, 66)	<0.001
**Male**	366 (57.1)	43 (58.1)	323 (57.0)	0.85
**Hypertension**	287 (44.8)	40 (54.1)	248 (43.7)	0.006
**Hyperlipidemia**	304 (47.3)	42 (56.8)	262 (46.2)	0.006
**Diabetes mellitus**	81 (12.6)	14 (18.9)	67 (11.8)	0.08
**BMI ≥30 kg/m**^**2**^^**†**^	231 (38.1)	20 (27.0)	211 (39.7)	0.036
**Tobacco use**	213 (33.2)	25 (33.8)	188 (33.2)	0.75
**Known CAD**^**†**^	92 (14.4)	19 (25.7)	73 (12.9)	0.013
**History of MI**^**†**^	66 (10.3)	7 (9.5)	59 (10.4)	0.44
**Prior revascularization**	85 (13.3)	15 (20.3)	70 (12.4)	0.06

* Continuous variables are given as median (25^th^, 75^th^ percentile).

^**†**^ BMI = body mass index; CAD = coronary artery disease; MI = myocardial infarction.

### ExECG findings

Table 2 shows the ExECG findings in the entire cohort and subdivided by presence or absence of aVR elevation ≥1mm. Although the median METs achieved were the same, the higher 75^th^ percentile for patients without aVR elevation led to a significant difference. There were no differences in maximum heart rate, maximum systolic blood pressure or rate pressure product between the two groups. The cohort with aVR elevation had a >2-fold increase in the incidence of angina during testing and an 8-fold increase in ST-depression ≥1mm. When these data are combined with exercise workload, the median Duke Treadmill Score (DTS) was significantly lower in the group with ≥1mm aVR elevation with moderate risk at 2.0 versus a low-risk score of 9.0 for those without aVR elevation ≥1mm. There was also a higher percentage of patients with a moderate or high-risk DTS in the subgroup with aVR elevation (p<0.001, Table 2). Other high-risk ST-changes were more common in the aVR elevation subgroup, including lead V1 ST-elevation and the combination of V1 ST-elevation and V5 ST-depression.

**Table 2:** Exercise and electrocardiographic findings stratified by presence or absence of ≥1mm aVR elevation.

Characteristic	Entire Cohort (n (%))	aVR Elevation ≥1mm (n (%))	No aVR Elevation (n (%))	P-value
**Patient Number**	641	74	567	-
METs Achieved*^,^^**†**^	10.1 (8.0, 12.0)	10.1 (8.0, 11.0)	10.1 (8.0, 12.0)	0.006
**Duke Treadmill Score**^**†**^	9.0 (6.0, 10.0)	2.0 (-2.0, 7.5)	9.0 (6.0, 11.0)	<0.001
Low Risk (DTS ≥5)*	494 (77.1)	31 (41.9)	463 (81.7)	<0.001
** Intermediate Risk**	119 (18.6)	36 (48.7)	83 (14.6)	
(DTS -10 to -4)*				
High Risk (DTS <-10)*	28 (4.4)	7 (9.5)	21 (3.7)	
Maximum Heart Rate (beats/min)*^,^^**†**^	160 (146, 171)	156 (142, 171)	160 (146, 173)	0.67
**Maximum Systolic Blood Pressure (mmHg)**^**†**^	186 (167, 203)	197 (166, 212)	185 (167, 201)	0.13
**Rate Pressure Product (x10**^**3**^**)**^**†**^	29.4 (25.4, 32.5)	30.0 (25.2, 33.3)	29.2 (25.5, 32.3)	0.35
**Angina During Testing**	68 (10.6)	16 (21.6)	52 (9.2)	0.001
**ST-Depression ≥1mm in any lead other than aVR**	88 (13.7)	45 (60.8)	43 (7.6)	<0.001
**V5 ST-depression ≥1mm**	40 (6.2)	33 (44.6)	7 (1.2)	<0.001
**V1 ST-elevation ≥1mm**	57 (8.9)	31 (41.9)	26 (4.6)	<0.001
**V1 ST-elevation and V5 ST-depression ≥1mm**	16 (2.4)	16 (21.6)	0 (0)	<0.001

* DTS = Duke Treadmill Score; ECG = electrocardiogram; MET = metabolic equivalent; min = minute.

^**†**^ Continuous variables are given as median (25^th^, 75^th^ percentile).

### Imaging results

**Fig 3:**
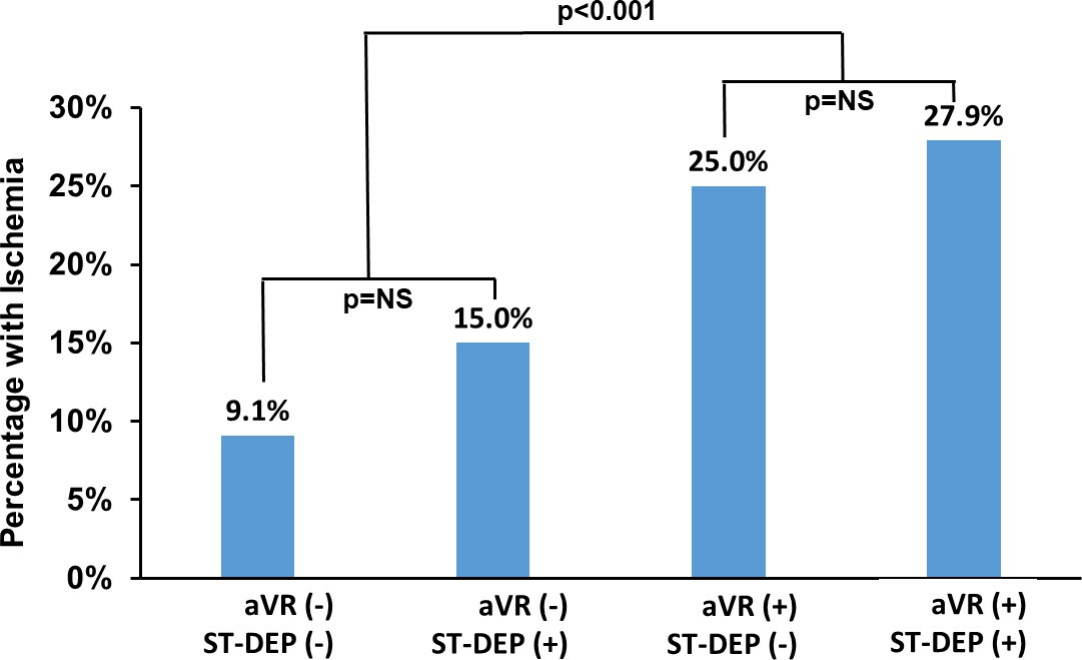
Prevalence of ischemia on SPECT myocardial perfusion imaging by the presence or absence of ≥1mm ST-depression in all leads and ≥1mm elevation in lead aVR. There was a significant difference in prevalence by presence of absence of aVR elevation, but no significant difference by ST-depression within each aVR subgroup.

Of the 641 in the final study cohort, 104 patients underwent ExECG only (16.2%), while echocardiography was performed in 184 (28.7%), and 353 (55.1%) had concomitant SPECT MPI. Ischemia in at least one segment was present in 63 of the 537 (11.7%) who had imaging. The prevalence of ischemia in patients with ≥1mm aVR elevation was 26.9% versus 9.6% in those without ≥1mm aVR elevation (p<0.0001). Of the patients who underwent SPECT MPI, the rate of ≥10% LV ischemia was 14.3% in those with ≥1mm aVR elevation versus 2.3% in patients without ≥1mm aVR elevation (p<0.0001). There were 3 patients with ≥20% LV ischemia, all of whom had ≥1mm aVR elevation (p = 0.003). The percentage of subjects with any ischemia subdivided by aVR elevation and ST-depression ≥1mm are provided in Fig 3. The prevalence of ischemia within each aVR group is not significantly different based on the presence of ST-depression, but there is a significant lower prevalence of ischemia in those with aVR elevation versus no aVR elevation.

### Angiographic results and early revascularization

There were 14 patients with obstructive disease on invasive coronary angiography, 11 of whom (78.6%) had ≥1mm aVR elevation. Of the 8 patients who underwent early revascularization, 7 (87.5%) had aVR elevation ≥1mm. The rates of early revascularization in those with and without ≥1mm aVR elevation were 10.9% and 0.19%, respectively (p<0.001). We found the negative predictive value of ≥1mm aVR elevation for early revascularization to be 99.8%. The positive predictive value of ≥1mm aVR elevation for early revascularization was still low at 9.5%, given the low prevalence of this outcome. Sensitivity and specificity were 87.5% and 89.4%, respectively.

Univariable predictors of early revascularization were METS achieved (Odds Ratio (25th, 75th Percentile) 1.4 (1.1, 2.0), p = 0.019), Duke Treadmill Score (O.R. 1.4 (1.2, 1.6), p<0.001) and ≥1mm aVR elevation (O.R. 59.1 (7.1, 488.0), p<0.001). Both DTS (p = 0.006) and aVR elevation (p = 0.019) remained significant predictors in the multivariable logistic regression model, which was highly predictive of early revascularization with a C-statistic of 0.96. Lead aVR elevation had incremental diagnostic value over the Duke Treadmill Score (p = 0.008).

**Fig 4:**
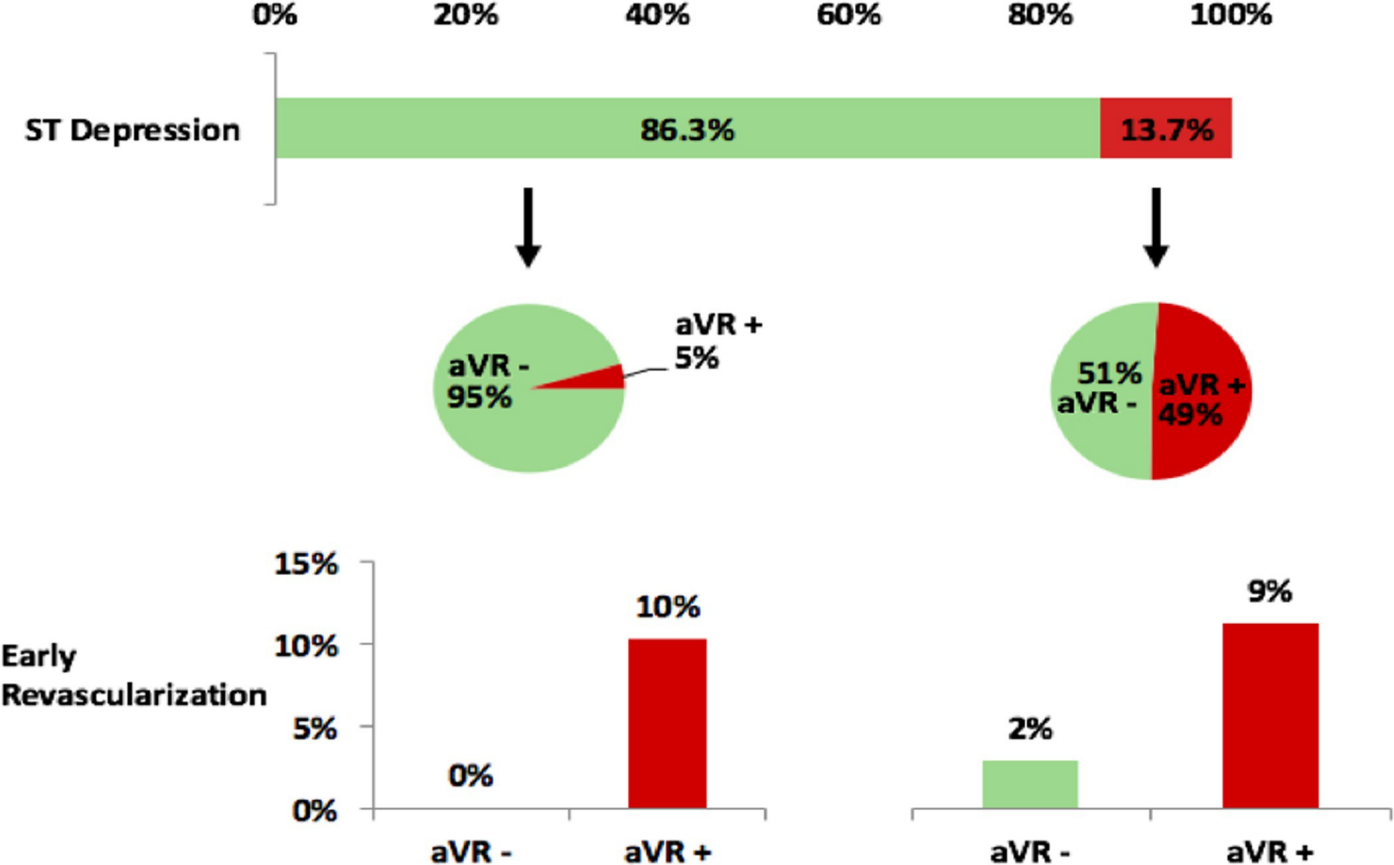
Lead aVR results in those with and without ST-depression and rates of early revascularization. In those without ST-depression, the rate of early revascularization was significantly higher in those with aVR elevation (p<0.001). In those with ST-depression, the subgroup with aVR elevation had a higher rate that was non-significant (p = 0.18) possibly due to reduced power from low numbers with ST-depression.

Fig 4 shows the breakdown of aVR values in patients with and without ST-depression and the correlating rates of early revascularization. Of the 553 subjects without ST-depression, 524 (94.8%) did not have aVR elevation. No subjects without ST-depression or aVR elevation had early revascularization. In the remaining 29 subjects with aVR elevation, 3 had early revascularization (10.3%). This difference was significant with p<0.001. In the 88 subjects with ST-depression, 45 (51.1%) had aVR elevation. Four of these subjects had early revascularization (8.9%) versus 1 of the 43 subjects without aVR elevation (2.3%). This difference in rates was not statistically significant, but the comparison is limited by the small subgroup sample size.

### Prevalence and predictors of cardiac events

Follow up data was available in 561 of the 641 patients (88%). The median follow-up time was 4.0 years (25^th^, 75^th^: 2.8, 4.4 years). A comparison of characteristics between those with and without follow-up available reveals a higher rate of hypertension (p<0.001) and CAD (p = 0.035) in those with follow-up available. There were no differences in age, gender, diabetes, hyperlipidemia, tobacco use, history of MI, or prior revascularization.

**Fig 5:**
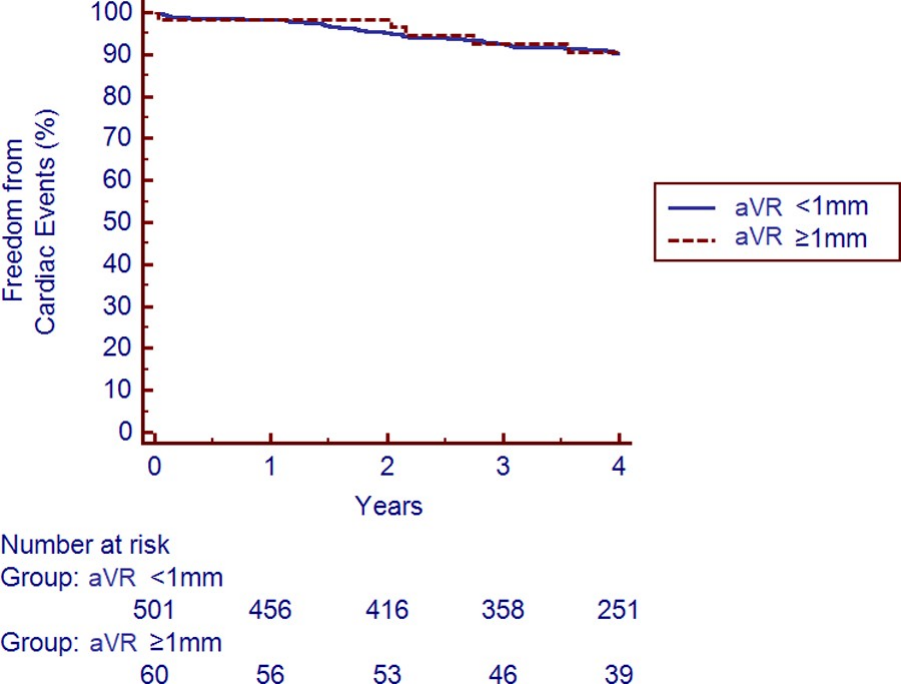
Kaplan-Meier survival analysis for freedom from cardiac events stratified by ≥1mm of aVR elevation. The presence or absence of ≥1mm elevation in lead aVR did not increase the risk of the composite outcome of cardiac death, nonfatal myocardial infarction, or late revascularization over a median 4.0 years of follow-up (p = 0.94).

There were 51 cardiac events over the entire follow-up period. The total number of events and yearly rates subdivided by aVR elevation are shown in Table 3. aVR elevation was not predictive of the occurrence of all cardiac events nor of any individual event assessed. The Kaplan Meier survival analysis revealed no difference in freedom from cardiac events based on aVR elevation after 4 years of follow-up (p = 0.94), as shown in Fig 5.

**Table 3:** Total incidence of cardiac events and annualized event rates stratified by presence or absence of ≥1mm aVR elevation in the 561 subjects with follow-up available.

Event	aVR Elevation ≥1mm (n (%/year))	No aVR Elevation (n (%/year))	P-value
**Patient number**	60	501	-
**Cardiac events**	6 (2.8)	55 (2.6)	0.80
**Cardiac death**	0 (0)	1 (0.1)	0.89
Nonfatal MI*	3 (1.4)	14 (0.8)	0.41
Cardiac Death or nonfatal MI*	3 (1.4)	15 (0.9)	0.43
**Late revascularization**	2 (0.9)	25 (1.5)	0.76

* MI = myocardial infarction.

In contrast, the DTS did predict total cardiac events. The 126 subjects with a moderate-risk score had 21 cardiac events (5.0%/yr), while the 412 subjects with a low-risk score had only 30 events (2.1%/year), p = 0.007. Only one subject had a high-risk score and did not have a subsequent event.

Cox proportional hazards univariable analysis for cardiac events identified the following significant predictors: age, hypertension, diabetes, known CAD, prior MI, prior revascularization, ischemia, and DTS. We entered these factors into a multivariable model with the results in Table 4. We chose to include prior MI rather than known CAD or prior revascularization as these had lower univariable X^2^ values and provide similar information. aVR elevation did not predict cardiac events (p = 0.35).

**Table 4:** Multivariable Cox proportional hazards analysis for total cardiac events.

Predictor	Χ^2^	Hazard Ratio	95% Confidence Interval	P-value
Prior MI*	11.8	3.25	1.66–6.39	0.004
**Ischemia**	9.3	2.80	1.45–5.43	<0.001
**Duke Treadmill Score (1 point increase)**	6.9	0.93	0.88–0.98	0.008
**aVR ≥ 1mm**	0.9	0.62	0.22–1.70	0.348

* MI = myocardial infarction.

Incremental X^2^ analysis of a Cox model for the prediction of cardiac events showed a significant improvement adding the DTS to clinical variables (history of MI, age, diabetes) with a X^2^ of 32.0, p = 0.032. Ischemia also brought incremental prognostic value (incremental X^2^ 12.2, p = 0.004). However, ≥1mm aVR elevation did not add incremental predictive value for the prediction of total cardiac events in models with (p = 0.91) or without (p = 0.40) ischemia.

## Discussion

Multiple prior studies have found a relationship between ST-elevation in lead aVR and the presence of obstructive CAD on invasive coronary angiography and adverse cardiac outcomes across a variety of patient subsets. In the setting of acute coronary syndrome, ST-segment elevation in lead aVR has been shown to be an indicator of left main or left anterior descending coronary artery disease [8, 20]. A meta-analysis of 22,740 patients identified the extent of aVR elevation to be one of the most powerful predictors of left main or 3-vessel disease in this setting [21].

aVR elevation, alone and coupled with other ECG changes, has also been studied well in patients referred for coronary angiography after ExECG. The combination of aVR elevation and ST depression in V5 during ExECG has been highly correlated with obstructive CAD in patients referred for coronary angiography, occurring in 99% of the cohort studied by Michaelides et al. [22]. The majority of subjects had LAD involvement and 92% had multivessel disease. In a separate cohort with single vessel obstructive CAD, this same group found a high likelihood of LAD stenosis, typically proximal, in patients with aVR elevation and V5 ST-depression [7]. Even without concomitant ECG changes, aVR elevation identifies high risk disease. In a cohort of 454 patients who underwent stress testing and coronary angiography, aVR elevation was the strongest predictor of obstructive left main or ostial LAD CAD with a predictive accuracy of 80% and a 45% post-test probability [10]. In this same study, aVR elevation was noted in 39% of patients with multivessel disease and 27% of patients with two vessel CAD–both in the absence of left main or proximal LAD disease. Therefore, lead aVR elevation does not appear to be exclusively found in patients with left main or proximal LAD disease, but rather may be found in patients with a higher burden of CAD [10].

However, there have been few published studies to date examining the rate of aVR elevation in the general population undergoing ExECG. Prior research has predominately been limited to patients who have undergone coronary angiography. Limiting analysis to this subset leads to selection bias and increases the likelihood of higher risk CAD. Multiple editorials have called for evaluation of “non-enriched populations” and thus identification of the “true specificity” of aVR elevation for significant CAD [11, 12]. A key component of evaluation in the general population is assessment of downstream cardiac events to ensure high risk patients are not underdiagnosed. Our analysis is one of the few to evaluate the prevalence and significance of aVR elevation in a general population undergoing ExECG with follow-up for events. Wagener and colleagues enrolled 1596 patients with suspected myocardial ischemia who were referred for bicycle stress testing with nuclear MPI. They found that 27% of their cohort had inducible myocardial ischemia and that major adverse cardiovascular events occurred in 33% of patients with aVR elevation and in 16% without [23].

The prevalence of aVR elevation in studies examining populations undergoing coronary angiography has been approximately 25% [10, 24]. In contrast, we found a much lower prevalence of 11.5% in a general population undergoing ExECG. This change in prevalence significantly effects test characteristics, especially the positive and negative predictive values. The prevalence of CAD is likely a strong contributor to this difference in aVR elevation prevalence. In the study by Michaelides et al., the prevalence of CAD was 87% [7]. This is much higher than the prevalence in a general population undergoing ExECG. The PROMISE cohort is representative of the general stress testing population and had a CAD prevalence of 10.7% in those randomized to a coronary evaluation by CT angiography [5].

In our analysis, aVR elevation predicted abnormal imaging results, with a 2.8-fold increased risk of ischemia. ST-depression did not significantly change the likelihood of ischemia when added to aVR elevation. The increased risk with aVR elevation was even more prominent for significant ischemia (≥10% of the LV), with a 6.2-fold increased likelihood. This is the level of ischemia shown to predict benefit with revascularization [25].

In the setting of an increased risk of significant ischemia, aVR elevation is associated with a 57-fold increased risk of early revascularization. This finding has important implications for the diagnostic evaluation of chest pain. ExECG without imaging is recommended as the initial test of choice in patients at intermediate risk for CAD who are able to exercise, but imaging is typically performed due to the low diagnostic accuracy of stress ECG alone [3, 26]. In our analysis of consecutive patients undergoing ExECG at a major academic medical center over a 7 month period, there were no occurrences of early revascularization in subjects without ST depression or aVR elevation (Fig 4). Moreover, the cohort without ST-depression or aVR elevation made up 81.7% of all subjects undergoing ExECG. This high negative predictive value may be beneficial in triaging patients for imaging following exercise stress testing. This analysis suggests that a strategy of exercise stress ECG with aVR evaluation could potentially limit the use of imaging in a substantial cohort of patients without missing higher risk patients in need of revascularization. Appropriately reducing imaging use in lower risk patients would reduce patient and societal costs, reduce radiation exposure, and aligns with patient-centered imaging principles [27, 28].

A substantial concern with a diagnostic strategy that does not include imaging is the potential of missing obstructive CAD that could lead to downstream cardiac events if not revascularized. However, it is important to note that the recently published ISCHEMIA trial (which included 5,179 patients) did not show an association with the degree of myocardial ischemia and all-cause mortality [29]. This study found that there was an association between the extent of CAD and all-cause mortality and MI [29]. In our study, we assessed events with a median follow-up of 4 years and observed a low rate of cardiac death and nonfatal MI in the entire cohort, including those subjects not receiving early revascularization. Moreover, there was no difference in events by presence or absence of ≥1mm aVR elevation, and aVR elevation did not predict cardiac events in the Cox model or add incremental prognostic value. The predictors of events in this study were all previously validated factors, such as the Duke Treadmill Score. In fact, the Duke Treadmill score has been shown to be a better predictor of CAD than ST depression alone and is effective for predicting major adverse cardiovascular events [30]. Therefore, the likelihood that aVR elevation is associated with high risk obstructive CAD in the absence of other high risk markers is low. This suggests that aVR elevation has initial diagnostic value for early catheterization but should not be used to predict long-term outcomes in patients undergoing ExECG.

The population selected for this study is more generalizable to the general population undergoing an evaluation for CAD, and thus the low prevalence of aVR elevation is more reflective of the true prevalence of disease. This study shows that while aVR elevation is a predictor of early revascularization in the population undergoing ExECG, it is not predictive of future cardiac events. Patients with aVR elevation were more likely to have ischemic ST changes in diagnostic leads, but more than 50% of patients with aVR elevation did not manifest these changes. Patients with aVR elevation tended to have more diagnostic signs on ECG suggestive of poor functional status, including angina. The nuclear and stress echo results are consistent with prior studies showing higher prevalence of ischemia in patients with aVR elevation. Finally, as has been demonstrated previously, patients with aVR elevation had a much higher rate of early revascularization. Additionally, the negative predictive value of aVR elevation for early revascularization was very high.

### Limitations

A limitation of this study is that it was a single-center analysis using data collected predominately retrospectively with the typical limitations that result. Follow-up data was collected prospectively, though data were not available in 12% of subjects. The characteristics in this subgroup did not differ significantly from those with follow-up available. The lack of randomization to diagnostic strategies and angiography could result in referral bias, but the lack of increased events suggests high risk disease was not missed. Finally, it should be emphasized that our analysis only applies to those undergoing ExECG rather than pharmacologic stress. Furthermore, the low numbers of enrolled patients with aVR elevation limits our evaluation of long-term cardiac events.

## Conclusions

In contrast to the close linkage between lead aVR elevation and high-risk obstructive CAD in studies of populations referred to invasive coronary angiography after ExECG, our analysis of the general population undergoing ExECG found a low positive predictive value for early revascularization. Nevertheless, aVR elevation has incremental diagnostic value and a high negative predictive value of 99.8% for early revascularization. No subjects without ST-depression or aVR elevation underwent early revascularization. Moreover, there was no increased risk of events over 4 years with aVR elevation. These findings suggest that aVR analysis should be routinely added to ST-analysis to improve diagnostic accuracy and potentially to improve strategies avoiding imaging in appropriate lower risk subgroups.

## Supporting information

S1 Data (XLSX)

S1 File (DOCX)
