# Supplementary material for: Lead aVR predicts early revascularization but not long-term events in patients referred for stress electrocardiography
Source: PLoS One. 2021 Apr 8;16(4):e0249779. doi: 10.1371/journal.pone.0249779 (PMC8032194; doi:10.1371/journal.pone.0249779)
Supplement: S1 File — (DOCX) [file pone.0249779.s002.docx]

**S1 File.**

Stress_Type

1=Exercise, 2=Pharmacologic

RBBB_Rest

0=None, 1=incomplete (QRS 100-119ms), 2=complete (QRS >=120ms)

LBBB_Rest

0=None, 1=incomplete (QRS 100-119ms), 2=complete (QRS >=120ms)

Bsl_AVR, V1, V5

Mm ST-depression (v5) or ST-elevation (AVR,V1) at rest – absolute value

Stress_Angina

0=none, 1=yes, 2=stopped test

Stress_SOB

0=none, 1=yes, 2=stopped test

PositiveStress

0=no, 1=Echo or MPI >1 (so positive)

V1Pos, V5Pos – either V1Max or V5max <= -1 or >= 1 =1 (positive), otherwise 0

History

Weight at time of study

BMI at time of study

Family history

0- negative; 1- positive

Digoxin

0- negative; 1- positive

Amiodarone

0- negative; 1- positive

History of congenital disease

0- negative; 1- positive

History of prior MI

0- negative; 1- positive

History of CABG

0- negative; 1- positive

History of atrial fibrillation

0- negative; 1- positive; 2- a-fib at time of study

History of CHF

1. negative; 1- positive

NYHA class (if applicable)

0, 1, 2, 3, 4

Pre-test likelihood of disease

Diamond and Forrester – Exercise Guidelines 2002

Gender

1- male; 2- female

Quality of angina

1. none; 1- typical; 2- atypical; 3-noncardiac

ACS at time of study

1. none; 1- unstable angina; 2- NSTEMI; 3- STEMI

Peak troponin elevation

Stress echo results

Stress LVEF

Study results

0- negative; 1- positive

Reversible perfusion defects

1- Anterior

2- Septal

3- Apical

4- Lateral

5- Inferior

6- Inferoposterior

Coronary angiography

-Specific stenoses

1- LAD

2- RCA

3- LCx

4- LAD and RCA

5- LAD and LCx

6- RCA and LCx

7- LAD, RCA, LCx (3VD)

-Revasc

0- negative; 1- positive

Endpoints

Death

0- negative; 1- positive

Date

Cause

0- non-cardiovascular; 1- cardiovascular

Myocardial infarction

1- NSTEMI; 2- STEMI

Date

Peak troponin elevation (if available)

Revasc

-Date

-Specific stenoses

1- LAD

2- RCA

3- LCx

4- LAD and RCA

5- LAD and LCx

6- RCA and LCx

7- LAD, RCA, LCx (3VD)

Follow-up

-Date of last known alive status

-Date of last known MI status
